# Impact of protein energy wasting status on survival among Afro-Caribbean hemodialysis patients: a 3-year prospective study

**DOI:** 10.1186/s40064-015-1257-3

**Published:** 2015-08-26

**Authors:** Lydia Foucan, Henri Merault, Fritz-Line Velayoudom-Cephise, Laurent Larifla, Cosmin Alecu, Jacques Ducros

**Affiliations:** Centre de dialyse AUDRA, Hôpital RICOU, Pointe-À-Pitre, Guadeloupe France; Département de Santé Publique, Equipe de recherche Epidémiologie Clinique et Médecine ECM/LAMIA, EA 4540, Centre Hospitalier Universitaire, Université des Antilles et de la Guyane, CHU de Pointe-à-Pitre, 97159 Pointe-à-Pitre, Guadeloupe France; Service de Néphrologie, Centre Hospitalier Universitaire, Pointe-à-Pitre, Guadeloupe France

**Keywords:** Afro-Caribbean patients, Body mass index, Hemodialysis, Mortality, Protein-energy wasting, Serum albumin

## Abstract

**Background:**

We assessed the prognostic value of protein-energy wasting (PEW) on mortality in Afro-Caribbean MHD patients and analysed how diabetes, cardiovascular disease (CVD) and inflammation modified the predictive power of a severe wasting state.

**Method:**

A 3-year prospective study was conducted in 216 patients from December 2011. We used four criteria from the nomenclature for PEW proposed by the International Society of Renal Nutrition and Metabolism in 2008: serum albumin 38 g/L, body mass index (BMI) ≤23 kg/m^2^, serum creatinine ≤818 µmol/L and protein intake assessed by nPCR ≤0.8 g/kg/day. PEW status was categorized according the number of criteria. Cox regression analyses were used.

**Results:**

Forty deaths (18.5 %) occurred, 97.5 % with a CV cause. Deaths were distributed as follows: 7.4 % in normal nutritional status, 13.2 % in slight wasting (1 PEW criterion), 28 % in moderate wasting (2 criteria) and 50 % in severe wasting (3–4 criteria). Among the PEW markers, low serum albumin (HR 3.18; P = 0.001) and low BMI (HR 1.97; P = 0.034) were the most significant predictors of death. Among the PEW status categories, moderate wasting (HR 3.43; P = 0.021) and severe wasting (HR 6.59; P = 0.001) were significant predictors of death. Diabetes, CVD, and inflammation were all additives in predicting death in association with severe wasting with a strongest HR (7.76; P < 0.001) for diabetic patients.

**Conclusions:**

The nomenclature for PEW predicts mortality in our Afro-Caribbean MHD patients and help to identify patients at risk of severe wasting to provide adequate nutritional support.

**Electronic supplementary material:**

The online version of this article (doi:10.1186/s40064-015-1257-3) contains supplementary material, which is available to authorized users.

## Background

Individuals from French oversea territories experience a higher incidence of end stage renal disease (ESDR) compared with continental French individuals: with 246/million inhabitants in Guadeloupe, a French Caribbean island, vs 149/million inhabitants in continental France (Briancon et al. 2011). High frequencies of traditional risk factors, which affect survival, were also reported in this population having a majority of patients of African ancestry (Foucan et al. 2000, 2012). In patients on maintenance hemodialysis (MHD), cardiovascular disease, diabetes mellitus and inflammation contribute to the high death rate (Goodkin et al. 2004). Uremic malnutrition, also called, protein energy wasting (PEW), corresponding to a decrease in energy and body protein, was consistently associated with mortality in different populations (Kovesdy and Kalantar-Zadeh 2009; Noori et al. 2011; Streja et al. 2011) and appears as the strongest risk factor for adverse outcome and death (Kovesdy and Kalantar-Zadeh 2009).

In a nomenclature for PEW proposed by the International Society of Renal Nutrition and Metabolism (Fouque et al. 2008), several parameters among four established categories (biochemical criteria; body mass and composition, muscle mass and dietary intakes) are indicative of PEW in individuals with kidney disease. Ethnic disparities were reported for the PEW biochemical markers (Noori et al. 2011) but also for muscle mass (Gallagher et al. 1985; Kramer et al. 2008). Recently, a score derived from this nomenclature predicted survival in the European ARNOS prospective dialysis cohort but, the need to evaluate the predictive ability of this PEW classification for mortality in non-Western populations was highlighted (Moreau-Gaudry et al. 2014).

In the present study we assessed the prognostic value of PEW on mortality in Afro Caribbean MHD patients and analyzed how several factors of interest modified the predictive power of a severe wasting state.

## Subjects and methods

### Patient population

In the present study, we included patients who underwent MHD treatment for more than 3 months and evaluated in December 2011 in the AUDRA centre (one of the dialysis facilities in the island of Guadeloupe, France). They were prospectively followed up from December 31, 2011, to December 31, 2014.

Standard dialysis treatment consisted of three weekly sessions using bicarbonate buffer and synthetic high flux membrane. Weekly dialysis time was 12 h in 83 % of patients. Dialysis dose delivery was estimated from the urea Kt/V (urea clearance over time).

### Data collection

Demographic and clinical data such as age, sex, dialysis vintage (time on HD), anthropometric parameters, cardiovascular risk factors, history of cardio vascular events and nutritional supplementation were recorded. Body mass index (BMI) in kg/m^2^ was calculated as dry weight divided by height squared.

Dialysis vintage was defined as the duration of time between the first day of HD treatment and December 31, 2011. Interdialytic weight gain (IDWG) was calculated as the difference between predialysis body weight and preceding postdialysis body weight during the month preceding the start of the study (December 2011) and the month preceding the end of follow-up for each patient. The mean value of 12 IDWG during the month was taken into account.

Dates and primary causes of death were recorded. The survival time was defined as the number of days between December 31, 2011 and the date of death or the date of censoring due to loss to follow-up (transfer to another dialysis centre or kidney transplantation) or the end of the follow-up at December 31, 2014.

### Laboratory measures

All laboratory values were measured by automated and standardized methods. Predialysis samples were collected for serum albumin, transthyretin, creatinine and highly sensitive C-reactive protein (hsCRP) measurements. Serum albumin, transthyretin and creatinine concentrations were determined.

The normalized protein catabolic rate (nPCR) (Aparicio et al. 1999; Daugirdas 1989) was used to assess the dietary protein intake with the following formula.

$${\text{nPCR}} \, = \,(0.0 1 3 6 { } \times {\text{ F}}) + 0. 2 5 1 {\text{ in g}}/{\text{kg per day}}$$

where F is equal to Kt/V × ([predialysis BUN + postdialysis BUN]/2).

### Definition of clinical factors and events

*PEW* One component in each of the four categories of the wasting syndrome (Fouque et al. 2008) were retained: serum albumin ≤38 g/L, BMI ≤23 kg/m^2^, SCr ≤25th percentile (µmol/L/m^2^) and nPCR ≤0.8 g/kg/day. Slight wasting was defined when one criteria for PEW was present, moderate wasting when two criteria for PEW was present, and severe wasting in presence of three or four criteria for PEW.

- Inflammation was defined as a serum concentration of CRP of >5 mg/L
- Cardiovascular disease (CVD) was defined as pre-existing coronary artery disease or stroke. Pre-existing CV complications included, coronary event and stroke occurred before December 2011.
- Weight loss: was defined as −5 % over 3 months (Fouque et al. 2008).

Outcome data considered as death of all causes were obtained from medical record.

### Statistical methods

Data are presented as percentages for categorical variables and as means ± standard deviations (SD) for continuous variables. Statistical methods included the *χ*^2^, ANCOVA adjusted for age and sex.

Multivariate Cox proportional hazards models were performed to analyze survival and to study the associations of PEW markers and PEW status as independent variables with death of all causes. The PEW markers (serum albumin ≤38 g/L, BMI ≤23 kg/m^2^, SCr ≤25th percentile in µmol/L and nPCR ≤0.8 g/kg/day) and the PEW status (normal nutritional status, slight wasting, moderate wasting and severe wasting) were studied separately. The variables of adjustment were: age (<60 or ≥60 years), gender, dialysis vintage (<5 or ≥5 years), diabetes, pre-existing CVD and hsCRP (<5 or ≥5 mg/L).

We also explored the potential additive association between severe wasting and three other risk factors of death in HD patients (pre existing CVD, diabetes and inflammation).

Hazard ratios (HR) and 95 % confidence interval (95 % CI) were provided. Significance was set at P < 0.05.

Statistical analyses were performed by using IBM-SPSS statistical software package version 21 (IBM, Armonk, NY, USA).

## Results

Overall, 216 patients were included in the analysis. The population was 56.5 % male. All the patients had diuresis lower than 500 mL/day (i.e. no residual renal function). The relevant demographic, clinical, and laboratory data of the study patients are presented in Additional file 1: Table S1. Mean age at baseline was 60 years and median dialysis vintage was 6.4 years. Average Kt/V was 1.31. Inflammation (CRP >5 mg/L) was found in 42.6 % of patients.

The major comorbidities were hypertension (91 %), diabetes (37 %), obesity (17 %) and previous cardiovascular events (13.9 %). Inflammation (CRP >5 mg/L) was found in 42.6 % of the patients.

Diabetic patients were more likely to have previous CVD than non-diabetic patients (23.5 vs. 8.1 %; P = 0.002).

Thirty patients were censored: 22 following kidney transplantation and 8 following transfer to another HD centre.

Forty deaths (18.5 %) occurred during the 3-year follow up, 97.5 % with a CV cause and 2.5 % with a non CV cause.

Deaths were distributed as follow: 5/65 (7.7 %) in normal nutritional status, 10/80 (12.5 %) in slight wasting, 14/50 (28 %) in moderate wasting and 10/20 (50 %) in severe wasting.

The causes of death were stroke (37.5 %), myocardial infarction (32.5 %), acute mesenteric ischemia (15 %), cardiac insufficiency (12.5 %) and sepsis (2.5 %).

In comparing at baseline clinical, and laboratory data of patients who died and those who survived, at baseline, patients who died were more likely to be older (P < 0.001), to have diabetes (P < 0.001), a weight loss over 3 months (P < 0.001), previous cardiovascular disease (P = 0.006) and to have lower serum albumin (P < 0.001), lower serum creatinine values (P = 0.013) and higher frequency of inflammation (P = 0.014) (Additional file 1: Table S1).

Additional file 1: Table S2 details the frequencies of the nutritional markers and the nutritional status at baseline. Significant differences were noted between patients who died and who survived for the biochemical criteria (P < 0.001), body mass (P **=** 0.025) and for the surrogate of muscle mass (P **=** 0.015) but not for dietary intakes (P **=** 0.114). A normal nutritional status was more frequently noted in those who survived (P **=** 0.007) whereas moderate or severe wasting were more frequent in patients who died (P **=** 0.022 and P < 0.001, respectively). Nutritional supplement was more frequently prescribed in this last group (P < 0.001).

At the end of follow-up, 84.3 % of HD patients were dialyzed with an arterial venous fistula and 15.7 % with a central venous catheter (CVC). There was no relationship between the type of vascular access and the nutritional status (CVC 15.4 vs 15.9 %; P = 0.925 in subjects with normal nutritional status and in those having 1 or more nutritional markers, respectively).

Additional file 1: Table S3 presents the IDWG according to the presence/absence of the nutritional markers. The IDWG at the start of the study and at the end of follow-up were significantly lower in presence of nutritional markers than in their absence, except for serum albumin ≤38 g/L.

Additional file 1: Table S4 presents the adjusted HR (95 % CI) of death for PEW markers and for PEW status. Both models were adjusted for the following parameters: age ≥60 years, gender, dialysis vintage ≥5 years, diabetes, pre existing CVD, CRP >5 mg/L.

Concerning the models including the four PEW markers (model 1): the multivariate Cox regression showed, significant associations between age ≥60 years (P = 0.049), low BMI (P = 0.034), low serum albumin (P = 0.001) and mortality. The association was nearly significant for low serum creatinine (P = 0.051) but not significant for gender, diabetes, CRP >5 mg/L and low protein intake.

Concerning the model including the PEW status (model 2): significant associations were noted between diabetes (P = 0.034), moderate PEW (P = 0.021), severe PEW (P = 0.001) and mortality. The association was nearly significant for age (P = 0.062) and CRP ≥5 mg/L (P = 0.081) but not significant for gender, pre existing CVD and slight wasting.

**Fig. 1:**
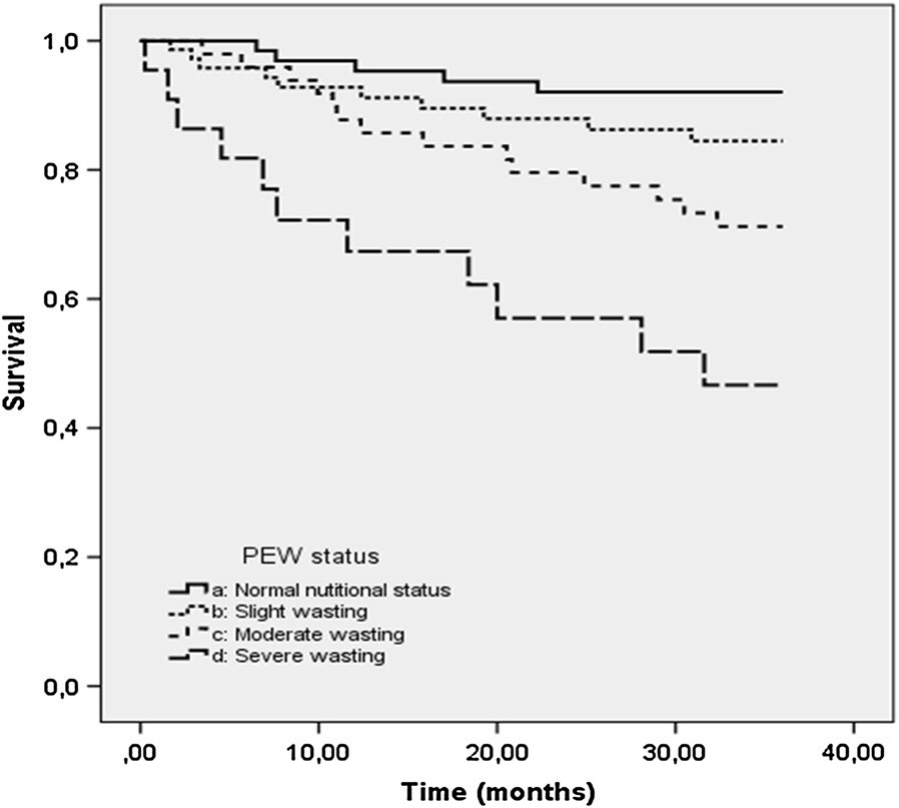
Survival curves according to protein-energy wasting status (*P* < 0.001, log-rank test)

The mean survival time [95 % confidence interval (CI)] was 31.6 (30.2, 32.9) months in the overall study population. This survival time was, respectively 23.5 (17.6, 29.5) months in patients who had a severe wasting; 30.5 (27.7, 32.3) months in those who had a moderate wasting; and 32.5 (30.3, 34.6) months in those who had a slight wasting; and 34.2 (32.6, 35.8) months in those who had a normal nutritional status. These times were significantly different between the four groups (P < 0.001, log-rank test). There was no statistical significant difference in survival between patients with slight wasting and those with normal nutritional status (P = 0.18, log-rank test). The survival curves according to PEW status are shown in Fig. 1.

**Fig. 2:**
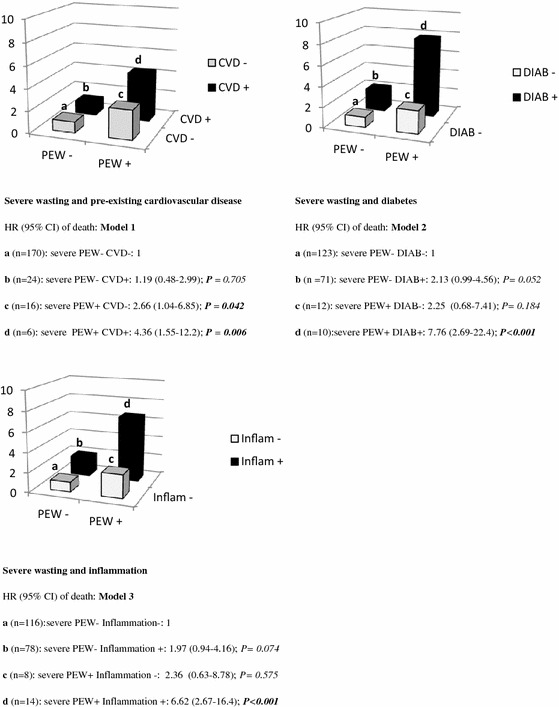
Severe wasting (at least three criteria for PEW) and additive markers (diabetes, previous cardiovascular event and inflammation) as predictors of all causes of mortality. *PEW* protein energy wasting, *CVD* previous cardiovascular disease, *Inflam* inflammation. Patients were divided into four groups according to PEW (presence/absence) and the additive factor (presence/absence). Model 1: adjustment for age, sex, diabetes and inflammation. Model 2: adjustment for age, sex, previous cardiovascular event and inflammation. Model 3: adjustment for age, sex, diabetes and previous cardiovascular event

Since cardiovascular disease, diabetes mellitus and inflammation contribute to the high death rate in HD patients, we evaluated the potential additive effects of these co morbid factors on severe wasting (Fig. 2). These factors are all additives in predicting death in our HD patients but, diabetes appears as the strongest predictive factor of death in association with severe wasting. In comparing to patients without severe wasting and without diabetes, patients who had these both factors exhibited a HR of death of 7.76 (P < 0.001) whereas this HR was 2.25 (P < 0.184) for patients having a severe wasting but no diabetes and 2.13 (P < 0.052) for those having diabetes but no severe wasting.

## Discussion

In this first study examining mortality according to nutritional status in a cohort of 216 Afro-Caribbean MHD patients who were followed for up to 3 years, we found that a lower BMI and lower serum albumin levels predicted worst survival. We also found a strong association between severe wasting status (three or more PEW criteria) and mortality. Pre existing cardiovascular disease, diabetes and inflammation incremented the risk of death associated with severe wasting, showing the contribution of all these factors to the high morbidity and mortality in patients with end-stage renal disease.

The nutritional and metabolic derangements observed in advanced chronic kidney disease cannot be attributed to any one single factor but, a common feature is the high rate of protein degradation (Marcelli et al. 2015). In the present study, we used four parameters among the established categories proposed by the International Society of Renal Nutrition and Metabolism to identify PEW (Fouque et al. 2008). Body mass index ≤23 kg/m^2^, serum albumin ≤38 g/L, SCr ≤25th percentile (µmol/L/m^2^) and nPCR ≤0.8 g/kg/day were the selected criteria. Excepted daily protein intake, all these parameters were significantly lower, at baseline, in patients who died during the study period.

The frequency of weight loss at baseline (5 % over 3 months) was higher in patients who died than in the others (22.5 vs 4 %, P < 0.001). According to the expert panel, a weight loss of 5 % over 3 months might also be considered an indicator of PEW (Fouque et al. 2008). In addition our patients having a BMI ≤23 kg/m^2^ had a HR of death about 2 time higher than the others. By contrast to the general population, overweight is paradoxically associated with improve survival in MHD patients, at least in the short term (Park et al. 2013). While obesity provides some protection against malnutrition, a low BMI should be in part the reflect of undernutrition.

Among the four criteria selected for the diagnosis of PEW, a low serum albumin concentration was the strongest predictor of death in our study. Several studies demonstrated the relationship between this criteria and outcome (Lowrie and Lew 1990; Owen et al. 1993). Serum albumin is an indicator of visceral proteins stores and is affected by protein intake but also by several factors including inflammation and other co morbidities (Kaysen 1998). Some authors in a previous study reported that patients with a serum albumin level below 35 g/L had a relative mortality risk of 4 (Lowrie et al. 1995). This relative risk was 3.49 for serum albumin levels below 38 g/L in our study population.

Serum creatinine is considered as a surrogate of muscle mass, especially in HD patients without residual renal function (Kalantar-Zadeh et al. 2012) and all patients in our study had no residual renal function. In a previous study among HD patients, creatinine levels below 8.0 mg/dL (704 µmol/L) predicted higher mortality than those above 10 mg/dL (880 µmol/L) (Kalantar-Zadeh et al. 2010). Since there is no recognized threshold for low creatinine levels we chose in our study the 25th percentile of the whole value corresponding to 818 µmol/L (9.3 mg/dL). Patients who died during the study period had lower baseline creatinine levels than the others. Those who had Scr below 818 µmol/L had a hazard ratio of death two time higher than those with SCr above this threshold. It was reported that individuals of African ancestry have higher measures of muscle and lean mass with higher levels of SCr than whites (Noori et al. 2011) probably in relation with higher protein intake.

The mean daily protein intake at baseline was higher than 1 g/kg/day for our patient without significant difference in both groups. Protein catabolic rate below 1 g/kg/day was previously associated with increase morbidity and mortality (Acchiardo et al. 1983) and a minimal protein intake of 1.1 g/kg/day is recommended (Fouque et al. 2007). But, the criteria for protein intake in the nomenclature for PEW corresponded to values below 0.8 g/kg/day that was not associated with the risk of death in our study.

The IDWG was found significantly lower in presence of the nutritional markers than in their absence, except for serum albumin levels ≤38 g/L. IDWG is a surrogate of patient’s fluid and sodium intake but may also be an index of good appetite and nutritional status. A strong association between IDWG and nutritional markers has been reported with a potential risk of malnutrition in HD patients if the weight gain is low (Lopez-Gomez et al. 2005; Sezer et al. 2002) Sezer SRen Fail. Our results indeed show that subjects with insufficient protein intake (nPCR ≤0.8 g/kg/day) or with low BMI had lower IDWG than the others which argues for a probable better nutrition in HD patients with high IDWG.

Patients in MHD suffer from multiple traditional or non traditional risk factors that are associated with mortality, such as diabetes, cardio vascular disease and inflammation. The Cox proportional hazard modelling (Fig. 2) showed that these three co-morbidities evaluated were all additives in predicting mortality in presence of a severe wasting. In fact, for the association of each risk factor and severe wasting, the HR of death was higher than the addition of HR of the single factors. Diabetes mellitus (DM) is the leading cause of end-stage renal diseases in Guadeloupe (Foucan et al. 2000) as in many countries and appears, in the present study, as the strongest predictive factor of death in association with severe wasting.

Although the main cause of death was cardiovascular disease, the predictive value of diabetes was higher than that of pre-existing cardiovascular disease. A possible explanation is that diabetic condition is associated with several abnormalities such as inflammation, oxidative stress (Basta et al. 2004; Nath et al. 2013) prothrombogenic factors (Kirpichnikov and Sowers 2001), accelerated atherosclerosis (Foley and Parfrey 1998) that lead to cardiovascular complications and poor prognosis in HD patients. In fact, as previously reported (Racki et al. 2007), preexisting CVD were more prevalent in our MHD diabetic patients than in non diabetics. Inflammation is known as an important contributor to PEW in ESRD patients since pro‐inflammatory cytokines stimulate protein catabolism (Bistrian et al. 1992), reduce albumin synthesis and contribute to anorexia (Plata-Salaman 1998). Protein energy wasting is known to be frequent in diabetic dialysis patients (Malgorzewicz et al. 2004) and to interact with inflammation and CVD (de Mutsert et al. 2008). Finally, all these factors contribute to the high death rate in MHD patients.

Our study has some limitations including the fact that the findings are limited to a relatively small number of patients. In addition, data were obtained from a prevalent cohort and potential variations of the criteria during the study period were not taken into account. Another limitation was that, we used a low BMI as one of the criteria for PEW. But, lean body mass and fat mass were not measured whereas it was previously suggested that their influence on survival are different (Beddhu et al. 2003; Noori et al. 2010).

One strength of our study was that the potential confounding effect of residual renal function was excluded because all patients in the present study had no residual renal function and in this situation higher creatinine levels relate to larger muscle mass and lower mortality (Park et al. 2013; Kalantar-Zadeh et al. 2010). There was also no bias in relation with type of dialysis or dialysis membrane since dialysis modalities were identical for all the subjects. In addition the data were obtained in a homogenous ethnic group of patients and this is of importance since measures of serum creatinine and other nutritional markers may vary according to ethnics groups.

## Conclusion

In MHD patients, survival decreases with the increasing number of criteria for PEW and an additional deleterious effect on survival related to severe wasting was observed in patients with diabetes or cardiovascular disease or inflammation. The nomenclature and diagnostic criteria for PEW proposed by the International Society of Renal Nutrition and Metabolism predicts mortality in our Afro-Caribbean MHD patients and could help to identify patients at risk of severe wasting to provide them adequate nutritional support. However, our results highlight the need to implement and evaluate prevention strategies and management of malnutrition in MHD patients.

## Additional file

Additional file 1:
**Table S1.** Baseline demographic, clinical, and laboratory parameters in 216 maintenance hemodialysis patients at the start of the 3-year cohort study. **Table S2.** Baseline nutritional markers values and nutritional status (categorical) in 216 maintenance hemodialysis patients at the start of the 3-year cohort study. **Table S3.** Interdialytic weight gain according to nutritional status and nutritional markers in 216 hemodialysis patients. **Table S4.** Multivariate Cox proportional hazard models and death hazard ratios of 3-year mortality according to PEW markers and PEW status in 216 hemodialysis patients.
