# Supplementary material for: Impact of protein energy wasting status on survival among Afro-Caribbean hemodialysis patients: a 3-year prospective study
Source: Springerplus. 2015 Aug 26;4:452. doi: 10.1186/s40064-015-1257-3 (PMC4549366; doi:10.1186/s40064-015-1257-3)
Supplement: Additional file 1: — Table S1. Baseline demographic, clinical, and laboratory parameters in 216 maintenance hemodialysis patients at the start of the 3-year cohort study. Table S2. Baseline nutritional markers values and nutritional status (categorical) in 216 maintenance hemodialysis patients at the start of the 3-year cohort study. Table S3. Interdialytic weight gain according to nutritional status and nutritional markers in 216 hemodialysis patients. Table S4. Multivariate Cox proportional hazard models and death hazard ratios of 3-year mortality according to PEW markers and PEW status in 216 hemodialysis patients. [file 40064_2015_1257_MOESM1_ESM.docx]

**Table S1: Baseline demographic, clinical, and laboratory parameters in 216 maintenance hemodialysis patients at the start of the 3-year cohort study.**

|  |  |  |  |  | |  |
| --- | --- | --- | --- | --- | --- | --- |
|  |  |  |  | **Death during the study period** | | *p* |
|  |  | **N =216** |  | **NO**  **n = 176** | **YES**  **n = 40** |  |
| Age (years) |  | 60 ± 13.9 |  | 58.6 ± 13.7 | 68.8 ± 15.5 | ***<0.001*** |
| Sex (men) (%) |  | 56.5 |  | 58.6 | 47.5 | *0.204* |
| Dialysis duration (months) * |  | 77.0 ± 64.9 |  | 75 ± 65.5 | 84 ± 62.5 | *0.466* |
| Kt / V* |  | 1.31± 0.18 |  | 1.30 ± 0.17 | 1.33 ± 0.18 | *0.326* |
| Diabetes (%) |  | 37.5 |  | 31.8 | 62.5 | ***<0.001*** |
| Hypertension (%) |  | 91.2 |  | 90.9 | 92.5 | *0.748* |
| BMI (Kg/m^2^) * |  | 25 ± 5.1 |  | 25.6 ± 4.8 | 24.0 ± 6.2 | *0.082* |
| Obesity (%) |  | 17.1 |  | 17.6 | 15.0 | *0.692* |
| Weight loss: >5% over 3 months **(%)** |  | 7.5 |  | 4.0 | 22.5 | ***<0.001*** |
| CVD (%) |  | 13.9 |  | 10.8 | 27.5 | ***0.006*** |
| Serum transthyretin (mg/L)* |  | 358 ± 266 |  | 374 ± 288 | 288 ± 97 | *0.076* |
| Serum albumin (g/L) * |  | 39.6 ± 4.2 |  | 40.4± 3.4 | 36.7 ± 5.6 | ***<0.001*** |
| SCr(µmol/L) * |  | 1053 ± 326 |  | 1078 ± 327 | 943 ± 259 | ***0.013*** |
| nPCRg/kg/day* |  | 1.02 ± 0.24 |  | 1.03 ± 0.24 | 1.01 ± 0.28 | *0.770* |
| C-reactive protein > 5 mg/L |  | 42.6 |  | 38.6 | 60.0 | *0.014* |
| Central venous catheters |  | 15.7 |  | 13.6 | 25.0 | *0.08* |

Values in this table are column percentage for categorical variables and mean ± SD for quantitative variables

*ANCOVA adjusted for age and sex. BMI: body mass index. CVD: previous cardiovascular events. SCr: serum creatinine. BSA: body surface area. nPCR: normalized protein catabolic rate. Four selected items for the wasting syndrome (in bold): Serum albumin (g/dL) ≤ 3.8, Body mass index (kg/m^2^) ≤ 23, SCr/BSA (mmol/L/m^2^) ≤ 380, nPCR≤ 0.8 g/kg/day

**Table S2: Baseline nutritional markers values and nutritional status (categorical) in 216 maintenance hemodialysis patients at the start of the 3-year cohort study.**

|  |  |  |  | **Death during the study period** | |  | |
| --- | --- | --- | --- | --- | --- | --- | --- |
|  |  |  |  | **No** | **Yes** | *P* |  |
|  |  | **N =216** |  | **n = 176** | **n = 40** |  |  |
| ***PEW markers*** |  |  |  |  |  |  |  |
| BMI ≤ 23 Kg/m^2^(%) |  | 37.0 |  | 33.5 | 52.5 | ***0.025*** |  |
| Serum albumin ≤ 38 g/L (%) |  | 31.5 |  | 24.4 | 62.5 | ***<0.001*** | |
| Serum creatinine ≤ 818 µmol/L |  | 25.0 |  | 21.0 | 42.5 | ***0.005*** | |
| nPCR ≤ 0.8 g/kg/day (%) |  | 20.8 |  | 18.8 | 30.0 | *0.114* | |
| ***PEW status*** |  |  |  |  |  |  | |
| Normal nutritional status (%) |  | 30.1 |  | 34.1 | 12.5 | ***0.007*** | |
| Slight wasting (%) |  | 37.0 |  | 39.8 | 25.0 | *0.081* | |
| Moderate wasting (%) |  | 23.6 |  | 20.5 | 37.5 | ***0.022*** | |
| Severe wasting (%) |  | 9.3 |  | 5.7 | 25.0 | ***<0.001*** | |
| Nutritional supplement (%) |  | 18.5 |  | 13.1 | 42.5 | ***<0.001*** | |

Values in this table are column percentage

BMI: body mass index. nPCR, normalized protein catabolic rate.

Four selected items for the wasting syndrome (in bold): Serum albumin (g/dL) ≤ 3.8, Body mass index (kg/m^2^) ≤ 23, Serum creatinine (mmol/L/m^2^) ≤ 380, nPCR≤ 0.8 g/kg/day

**Table S3 Interdialytic weight gain according to nutritional status and nutritional markers in 216 hemodialysis patients.**

|  | **Normal nutritional status** | | | **BMI**  **≤ 23 Kg/m^2^** | | | **Serum albumin**  **≤ 38 g/L** | | | **Serum creatinine**  **≤ 818 µmol/L** | | | **nPCR**  **≤ 0.8 g/kg/day** | | |
| --- | --- | --- | --- | --- | --- | --- | --- | --- | --- | --- | --- | --- | --- | --- | --- |
|  | YES | NO | *P* | YES | NO | *P* | YES | NO | *P* | YES | NO | *P* | YES | NO | *P* |
| **N** | 65 | 151 |  | 80 | 136 |  | 68 | 148 |  | 54 | 162 |  | 45 | 171 |  |
|  |  |  |  |  |  |  |  |  |  |  |  |  |  |  |  |
| **IDWG (Kg)**  **December 2011** | 3.0 ± 0.8 | 2.6 ± 0.9 | ***<0.001*** | 2,4± 0.8 | 2.8 ± 1.0 | ***<0.001*** | 2.6 ± 1.0 | 2.7 ± 0.9 | *0.803* | 2.3 ±1.0 | 2.8 ± 0.9 | ***<0.001*** | 2.3 ± 0.9 | 2.7 ± 0.9 | ***0.009*** |
| **IDWG (Kg)**  **end**  **of follow-up** | 2.9 ± 1.0 | 2.2 ± 1.1 | ***<0.001*** | 2.1 ± 0.9 | 2.6 ± 1.1 | ***0.002*** | 2.2 ± 1.2 | 2.5 ± 1.1 | *0.080* | 2.1 ± 1.1 | 2.6 ± 1.1 | ***0.009*** | 2.1 ± 1.0 | 2.6 ± 1.1 | ***0.018*** |

IDWG: Interdialytic weight gain

**Table S4: Multivariate Cox proportional hazard models and death hazard ratios of 3-year mortality according to PEW markers and PEW status in 216 hemodialysis patients**

|  | **Adjusted HR (95% CI )**  **of death for PEW markers**  **Model 1** | | | |  |  | **Adjusted HR (95% CI )**  **of death for PEW status**  **Model 2** | | |  |
| --- | --- | --- | --- | --- | --- | --- | --- | --- | --- | --- |
|  | n |  | HR (95% CI) | ***P*** |  |  | n | HR (95% CI) | ***P*** |  |
| Sex (Women/Men) | 94 |  | 1.25 (0.64-2.47) | *0.414* |  |  | 94 | 1.16 (0.60-2.22) | *0.649* |  |
|  |  |  |  |  |  |  |  |  |  |  |
| Age ≥ 60 Y (Yes/No) | 114 |  | 2.18 (1.01-4.72) | ***0.049*** |  |  | 114 | 2.09 (0.97-4.53) | *0.062* |  |
|  |  |  |  |  |  |  |  |  |  |  |
| Dialysis vintage ≥ 5 Y (Yes/No) | 115 |  | 1.05 (0.53-2.05) | *0.897* |  |  | 115 | 0.95 (0.49-1.81) | ***0.866*** |  |
|  |  |  |  |  |  |  |  |  |  |  |
| Diabetes (Yes/No) | 81 |  | 1.92 (0.97-3.81) | *0.061* |  |  | 81 | 2.11 (1.06-4.20) | ***0.034*** |  |
|  |  |  |  |  |  |  |  |  |  |  |
| Previous CVD (Yes/No) | 30 |  | 1.78 (0.80-3.96) | *0.159* |  |  | 30 | 1.52 (0.6- 3.32) | *0.296* |  |
|  |  |  |  |  |  |  |  |  |  |  |
| CRP ≥ 5 mg/L(Yes/No) | 92 |  | 1.55 (0.78-3.06) | *0.207* |  |  | 92 | 1.78 (0.93-3.40) | *0.081* |  |
|  |  |  |  |  |  |  |  |  |  |  |
| ***PEW markers*** |  |  |  |  |  |  |  |  |  |  |
| BMI ≤ 23 Kg/m^2^ (Yes/No) | 80 |  | 1.97 (1.05-3.70) | ***0.034*** |  |  | __ | __ | __ |  |
| Albumin ≤ 38 g/L (Yes/No) | 68 |  | 3.18 (1.60-6.30) | ***0.001*** |  |  | __ | __ | __ |  |
| Scr≤ 818 µmol/L (Yes/No) | 54 |  | 1.98 (1.00-3.91) | *0.051* |  |  | __ | __ | __ |  |
| nPCR ≤ 0.8 g/kg/D (Yes/No) | 45 |  | 1.19 (0.56-2.56) | *0.645* |  |  | __ | __ | __ |  |
| ***PEW status*** |  |  |  |  |  |  |  |  |  |  |
| Normal nutritional status | __ |  | __ | __ |  |  | 65 | 1 |  |  |
| Slight PEW / *No PEW* | __ |  | __ | __ |  |  | 80 | 1.75 (0.59-5.20) | *0.316* |  |
| Moderate PEW / *No PEW* | __ |  | __ | __ |  |  | 51 | 3.43 (1.21-9.73) | ***0.021*** |  |
| Severe PEW / *No PEW* | __ |  | __ | __ |  |  | 20 | 6.59 (2.23-19.5) | ***0.001*** |  |
